# Synthesis and characterization of 3-methyl-6-[(propyn­yloxy)meth­yl]-1,4-dioxane-2,5-dione

**DOI:** 10.1107/S2056989017008581

**Published:** 2017-06-16

**Authors:** Igor Elkin, Thierry Maris, Patrice Hildgen

**Affiliations:** a Faculty of Pharmacy, Université de Montréal, 2900 Edouard-Montpetit Blvd, Montreal, Quebec, H3T1J4, Canada; b Department of Chemistry, Université de Montréal, 2900 Edouard-Montpetit Blvd, Montreal, Quebec, H3T1J4, Canada

**Keywords:** crystal structure, 3-methyl-6-propynyloxymethyl-1,4-dioxane-2,5-dione, lactide, synthesis

## Abstract

The number of known asymmetrically substituted hemilactides, important precursors for obtaining regular derivatives of polylactide polymers, is still limited and structural characterization of most of them is incomplete. In the title racemic 1,4-dioxane-2,5-dione derivative, C_9_H_10_O_5_, the hemilactide heterocycle exhibits a twist-boat conformation. The bulkier propynyloxymethyl group is in an axial position with a *gauche* conformation for the CH_2_–O–CH_2_–C segment. In the crystal, mol­ecules are linked by pairs of C—H⋯O hydrogen bonds, forming inversion dimers. The dimers are linked by further C—H⋯O contacts, forming a three-dimensional structure.

## Abstract 2

The synthesis of a new asymmetrically substituted hemilactide is reported and its structural analysis, including X-ray crystallographic data, is reported.

## Chemical context   

Cyclic dilactides, or hemilactides, close structural analogs of 1,4-dioxane-2,5-dione (glycolide) with methyl- or methyl­ene-containing substituents at the *sp* ^3^ C atoms, are the most important precursors for obtaining polylactide polymers, which are widely employed in biodegradable plastics and in the food and biomedical industries due to their intrinsic biocompatibility and biodegradability (Gerhardt *et al.*, 2006 ▸). Well-tuned architectures of substituted hemilactides lead to the creation of new polylactide materials with regular structures that allow clarification of polymer behaviour at the supra­molecular level, as well as achieving new useful properties (Fuoco *et al.*, 2016 ▸; Trimaille *et al.*, 2007 ▸; Zhang & Song, 2014 ▸). Nevertheless, the further development of the field is hampered by the fact that asymmetrically substituted hemilactides still constitute a very limited group of compounds, the structural characterization of most of which remains incomplete. In this context, the goal of the present study was to elaborate a reliable protocol for obtaining 3-methyl-6-[(propyn­yloxy)meth­yl]-1,4-dioxane-2,5-dione, **1**.
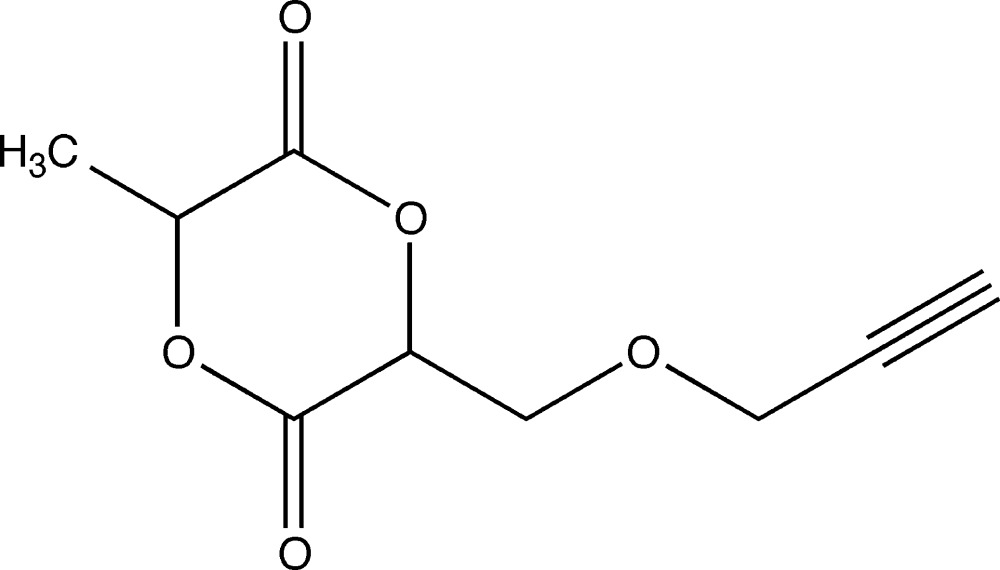


## Structural commentary   

**Figure 1:**
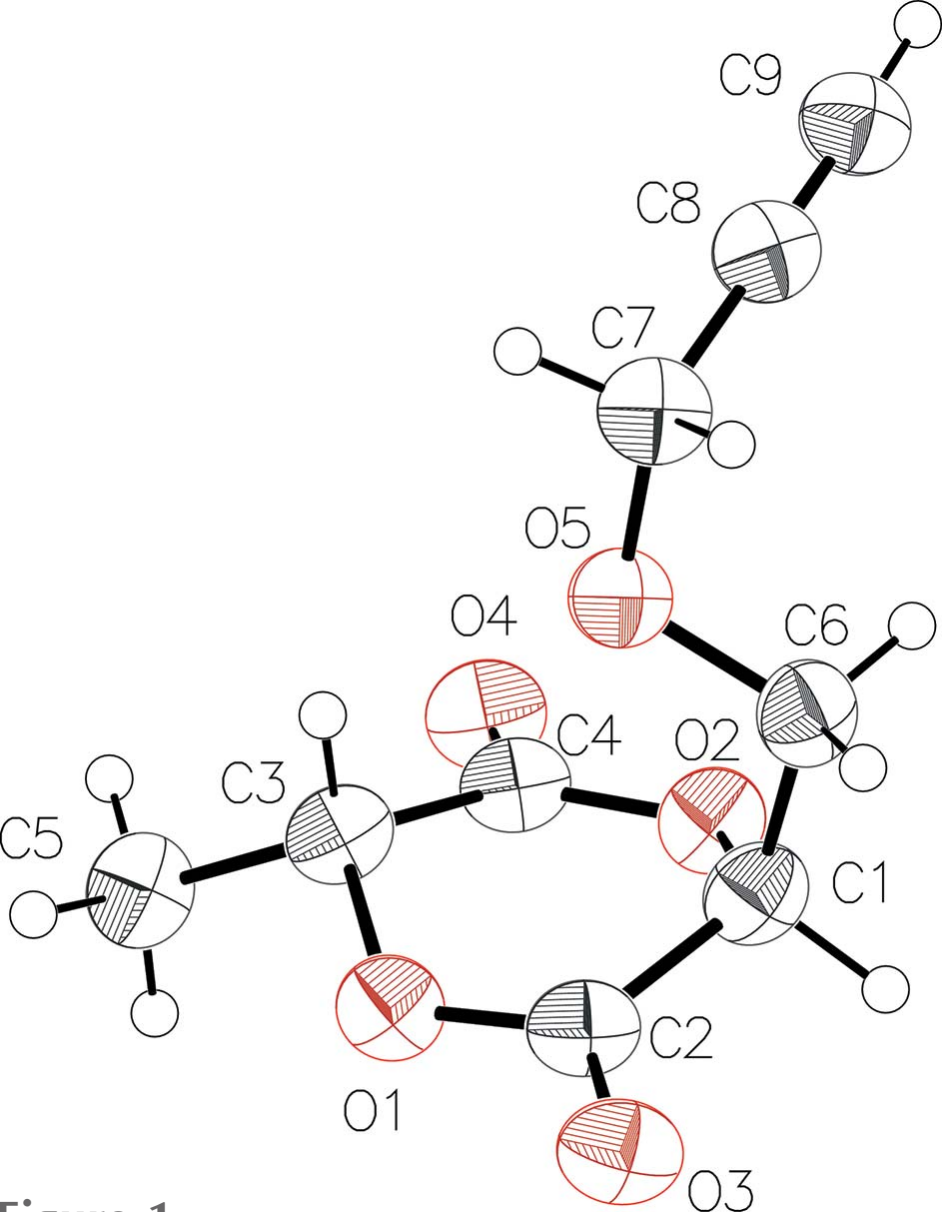
The atom-numbering diagram of the mol­ecule of **1**. C and O atoms are shown as displacement ellipsoids at the 50% probability level and H atoms are shown as spheres of arbitrary radius.

The mol­ecule of the final product (Fig. 1 ▸) possesses a 1,4-dioxane-2,5-dione six-membered ring, as well as the two different substituents, *i.e.* methyl and propynyloxymethyl groups, linked to atoms C1 and C3, respectively, determining the aimed architecture of **1**. In general, the bond lengths and angles are in normal ranges for organic carbohydrates. The hemilactide heterocycle exhibits a twisted boat conformation, where atoms C1, C2 and O1 are in one plane and atoms C1, C3, C4 and O2 are in another plane; the planes are inclined at a dihedral angle of 27.9 (2)°. The values of the observed puckering parameters [θ = 84.8 (3)° and φ = 308.2 (3)°] deviate slightly from those corresponding to an ideal boat conformation (θ = 90° and φ = 300°). Two stereocentres represented by the C1 and C3 atoms have opposite chirality, *i.e.* *R*,*S* (and *S*,*R* in the centrosymmetric counterpart), the substituents at which adopt a *trans* configuration with respect to the ring, by minimizing repulsive inter­actions. The bulkier propynyloxymethyl group is located above the ring, *i.e.* in the axial position with a *gauche* conf­ormation for the C6—O5—C7—C8 segment, at a dihedral angle of 71.3 (2)°. A similar conformation has been observed in *meso*-3,6-dipropargyloxymethyl-1,4-dioxane-2,5-dione (Zhang *et al.*, 2015 ▸).

## Supra­molecular features   

**Figure 2:**
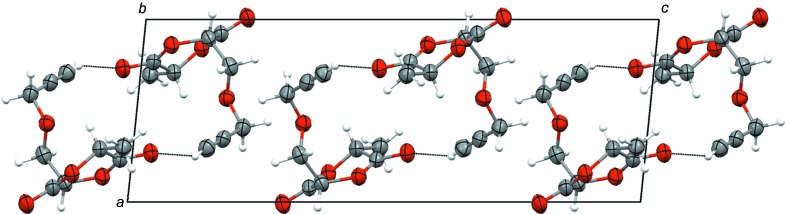
A view along the *b* axis of the crystal packing of **1**. Weak C—H⋯O contacts involving the acetylenyl H and ketone O atoms are shown as dotted lines.

In the crystal cell of **1** (Fig. 2 ▸), all the 1,4-dioxane-2,5-dione rings are located in parallel planes at a distance of approximately 2.0 Å, but do not tend to form mol­ecular stacks and organize the rings neither into columns, as reported for hemilactides bearing relatively small substituents, such as 3,6-dimethyl-1,4-dioxane-2,5-dione (van Hummel *et al.*, 1982 ▸) and 3-bromo-3,6-dimethyl-1,4-dioxane-2,5-dione and 3-methyl­ene-6-methyl-1,4-dioxane-2,5-dione (Fiore *et al.*, 2010 ▸), nor into supra­molecular formations where one half of the parallel plane is perpendicular to the other, as reported in the cases of 3-benzyl­oxymethyl-6-methyl-1,4-dioxane-2,5-dione (Kooijman *et al.*, 2005 ▸) and 3,6-diphenyl-l,4-dioxane-2,5-dione (Lynch *et al.*, 1990 ▸). In addition, the crystal packing shows some short C—H⋯O contacts (Table 1 ▸) leading to the pairwise mol­ecular binding, *i.e.* by hydrogen bonds. It is assumed that the packing is mostly determined by the contact involving the acid acetylenyl H9 and ketone O4 atoms (Fig. 2 ▸), analogous to the centrosymmetric inter­actions reported for symmetric *meso*-3,6-dipropargyloxymethyl-1,4-dioxane-2,5-dione (Zhang *et al.*, 2015 ▸). It is worthy of note that the unit cell contains no residual solvent-accessible voids.

## Database survey   

A search in the Cambridge Structural Database (Version 5.38 with two updates; Groom *et al.*, 2016 ▸) for pure and functionalized lactides (*i.e.* glycolides with one methyl substituent) returned 25 entries, including different lactide stereoisomers (Kooijman *et al.*, 2014 ▸; Fedushkin *et al.*, 2009 ▸; van Hummel *et al.*, 1982 ▸) and other derivatives (Zhang *et al.*, 2015 ▸; Fiore *et al.*, 2010 ▸; Kooijman *et al.*, 2005 ▸; Bolte *et al.*,1994 ▸; Lynch *et al.*, 1990 ▸).

## Synthesis and crystallization   

**Figure 3:**
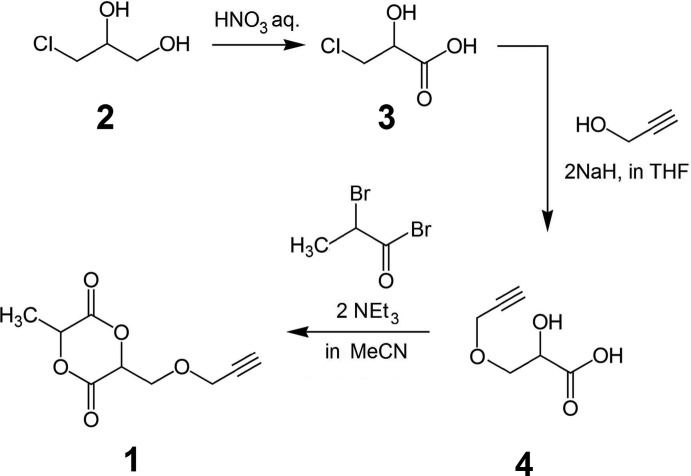
Scheme of the chemical synthesis of the title compound 3-methyl-6-[(propyn­yloxy)meth­yl]-1,4-dioxane-2,5-dione (**1**).

The desired product **1** was obtained from the initial *rac*-1-chloro­propane-2,3-diol (**2**) *via* a three-step pathway (see Fig. 3 ▸) inspired partly by general protocols (Bredikhina *et al.*, 2014 ▸; Trimaille *et al.*, 2004 ▸; Nagase *et al.*, 2008 ▸), comprising the oxidation of **2** to *rac*-3-chloro-2-hy­droxy­propanoic acid (**3**) followed by the etherification with propargyl alcohol to 2-hy­droxy-1-(propynyloxymeth­yl)propanoic acid (**4**) and the final double esterification of **4** with bromo­propyonyl bromide. The final purification of **1** was performed by auto-flash-chromatography on silica, using chloro­form as eluent to give, after evaporation under reduced pressure, a white crystalline solid (see supporting information for more details on the synthesis and structural characterization of the inter­mediate and final products).

## Refinement   

Crystal data, data collection and structure refinement details are summarized in Table 2 ▸. H atoms were located from Fourier difference maps and fully refined.

## Supplementary Material

Crystal structure: contains datablock(s) I. DOI: 10.1107/S2056989017008581/ff2148sup1.cif

Structure factors: contains datablock(s) I. DOI: 10.1107/S2056989017008581/ff2148Isup2.hkl

Details related to the synthesis, purification and characterization techniques used in the study. DOI: 10.1107/S2056989017008581/ff2148sup3.pdf

Supporting information file. DOI: 10.1107/S2056989017008581/ff2148Isup4.cml

CCDC reference: 1555000

Additional supporting information:  crystallographic information; 3D view; checkCIF report

## Figures and Tables

**Table 1:** Hydrogen-bond geometry (Å, °)

*D*—H⋯*A*	*D*—H	H⋯*A*	*D*⋯*A*	*D*—H⋯*A*
C3—H3⋯O4^i^	0.92 (2)	2.60 (2)	3.247 (3)	127.8 (16)
C3—H3⋯O5	0.92 (2)	2.49 (2)	3.033 (2)	118.1 (16)
C6—H6*B*⋯O3^ii^	0.99 (3)	2.47 (2)	3.369 (3)	151.0 (19)
C7—H7*A*⋯O3^iii^	0.98 (2)	2.66 (2)	3.627 (3)	169.1 (18)
C9—H9⋯O4^iv^	0.88 (3)	2.58 (3)	3.412 (3)	156 (2)

Symmetry codes: (i) 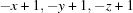; (ii) 
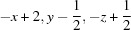
; (iii) 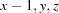; (iv) 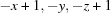.

**Table 2:** Experimental details

Crystal data	
Chemical formula	C_9_H_10_O_5_
*M*_r_	198.17
Crystal system, space group	Monoclinic, *P*2_1_/*c*
Temperature (K)	100
*a*, *b*, *c* (Å)	6.9774 (5), 6.8273 (5), 19.4895 (14)
β (°)	95.804 (3)
*V* (Å^3^)	923.66 (12)
*Z*	4
Radiation type	Ga *K*α, λ = 1.34139 Å
μ (mm^−1^)	0.65
Crystal size (mm)	0.11 × 0.08 × 0.08

Data collection	
Diffractometer	Bruker Venture Metaljet
Absorption correction	Multi-scan (*SADABS*; Krause *et al.*, 2015 ▸)
*T*_min_, *T*_max_	0.570, 0.752
No. of measured, independent and observed [*I* > 2σ(*I*)] reflections	19524, 2055, 1640
*R*_int_	0.073
(sin θ/λ)_max_ (Å^−1^)	0.652

Refinement	
*R*[*F* ^2^ > 2σ(*F* ^2^)], *wR*(*F* ^2^), *S*	0.063, 0.183, 1.05
No. of reflections	2055
No. of parameters	168
H-atom treatment	All H-atom parameters refined
Δρ_max_, Δρ_min_ (e Å^−3^)	0.35, −0.37

Computer programs: *APEX3* and *SAINT* (Bruker, 2016 ▸), *SHELXT* (Sheldrick, 2015*a* ▸), *SHELXL2016* (Sheldrick, 2015*b* ▸), *OLEX2* (Dolomanov *et al.*, 2009 ▸) and *Mercury* (Macrae *et al.*, 2008 ▸).

## supplementary crystallographic information

### Crystal data

**Table:**

C_9_H_10_O_5_	*F*(000) = 416
*M**_r_* = 198.17	*D*_x_ = 1.425 Mg m^−^^3^
Monoclinic, *P*2_1_/*c*	Ga *K*α radiation, λ = 1.34139 Å
*a* = 6.9774 (5) Å	Cell parameters from 9932 reflections
*b* = 6.8273 (5) Å	θ = 4.0–60.8°
*c* = 19.4895 (14) Å	µ = 0.65 mm^−^^1^
β = 95.804 (3)°	*T* = 100 K
*V* = 923.66 (12) Å^3^	Chunk, clear light colourless
*Z* = 4	0.11 × 0.08 × 0.08 mm

### Data collection

**Table:**

Bruker Venture Metaljet diffractometer	2055 independent reflections
Radiation source: Metal Jet, Gallium Liquid Metal Jet Source	1640 reflections with *I* > 2σ(*I*)
Helios MX Mirror Optics monochromator	*R*_int_ = 0.073
Detector resolution: 10.24 pixels mm^-1^	θ_max_ = 60.9°, θ_min_ = 4.0°
ω and φ scans	*h* = −9→9
Absorption correction: multi-scan (SADABS; Krause *et al.*, 2015)	*k* = −8→8
*T*_min_ = 0.570, *T*_max_ = 0.752	*l* = −25→25
19524 measured reflections	

### Refinement

**Table:**

Refinement on *F*^2^	Hydrogen site location: difference Fourier map
Least-squares matrix: full	All H-atom parameters refined
*R*[*F*^2^ > 2σ(*F*^2^)] = 0.063	*w* = 1/[σ^2^(*F*_o_^2^) + (0.1166*P*)^2^ + 0.2436*P*] where *P* = (*F*_o_^2^ + 2*F*_c_^2^)/3
*wR*(*F*^2^) = 0.183	(Δ/σ)_max_ < 0.001
*S* = 1.05	Δρ_max_ = 0.35 e Å^−^^3^
2055 reflections	Δρ_min_ = −0.37 e Å^−^^3^
168 parameters	Extinction correction: SHELXL2016 (Sheldrick, 2015b), Fc^*^=kFc[1+0.001xFc^2^λ^3^/sin(2θ)]^-1/4^
0 restraints	Extinction coefficient: 0.0045 (17)

### Special details

**Table:**

Experimental. X-ray crystallographic data for I were collected from a single crystal sample, which was mounted on a loop fiber. Data were collected using a Bruker Venture diffractometer equipped with a Photon 100 CMOS Detector, a Helios MX optics and a Kappa goniometer. The crystal-to-detector distance was 4.0 cm, and the data collection was carried out in 1024 x 1024 pixel mode.
Geometry. All esds (except the esd in the dihedral angle between two l.s. planes) are estimated using the full covariance matrix. The cell esds are taken into account individually in the estimation of esds in distances, angles and torsion angles; correlations between esds in cell parameters are only used when they are defined by crystal symmetry. An approximate (isotropic) treatment of cell esds is used for estimating esds involving l.s. planes.

### Fractional atomic coordinates and isotropic or equivalent isotropic displacement parameters (Å^2^)

**Table:**

	*x*	*y*	*z*	*U*_iso_*/*U*_eq_	
O1	0.8442 (2)	0.6986 (2)	0.38601 (7)	0.0450 (4)	
O2	0.8604 (2)	0.3277 (2)	0.44578 (7)	0.0445 (4)	
O3	1.0103 (2)	0.5925 (2)	0.30315 (8)	0.0528 (5)	
O4	0.7313 (2)	0.4446 (2)	0.53563 (7)	0.0500 (4)	
O5	0.5693 (2)	0.3519 (2)	0.32715 (7)	0.0433 (4)	
C1	0.8967 (3)	0.3454 (3)	0.37415 (10)	0.0442 (5)	
H1	1.029 (4)	0.277 (3)	0.3718 (12)	0.047 (6)*	
C2	0.9217 (3)	0.5552 (3)	0.35135 (10)	0.0427 (5)	
C3	0.7092 (3)	0.6521 (3)	0.43558 (10)	0.0413 (5)	
H3	0.591 (3)	0.624 (3)	0.4123 (11)	0.033 (5)*	
C4	0.7669 (3)	0.4688 (3)	0.47711 (10)	0.0412 (5)	
C5	0.6982 (4)	0.8289 (4)	0.48109 (12)	0.0490 (6)	
H5A	0.817 (4)	0.861 (4)	0.5017 (13)	0.050 (6)*	
H5B	0.656 (4)	0.940 (4)	0.4515 (14)	0.059 (7)*	
H5C	0.613 (4)	0.797 (4)	0.5153 (16)	0.061 (8)*	
C6	0.7425 (3)	0.2424 (3)	0.32707 (12)	0.0452 (5)	
H6A	0.725 (3)	0.111 (4)	0.3442 (12)	0.041 (6)*	
H6B	0.783 (3)	0.234 (3)	0.2801 (14)	0.047 (6)*	
C7	0.4081 (3)	0.2573 (3)	0.28966 (11)	0.0472 (5)	
H7A	0.299 (3)	0.349 (3)	0.2863 (11)	0.041 (6)*	
H7B	0.440 (3)	0.222 (3)	0.2426 (14)	0.047 (6)*	
C8	0.3455 (3)	0.0836 (3)	0.32598 (10)	0.0437 (5)	
C9	0.2952 (3)	−0.0550 (3)	0.35607 (12)	0.0476 (5)	
H9	0.252 (4)	−0.157 (4)	0.3776 (15)	0.060 (8)*	

### Atomic displacement parameters (Å^2^)

**Table:**

	*U*^11^	*U*^22^	*U*^33^	*U*^12^	*U*^13^	*U*^23^
O1	0.0527 (9)	0.0485 (8)	0.0346 (8)	−0.0021 (6)	0.0088 (6)	0.0033 (6)
O2	0.0478 (8)	0.0505 (8)	0.0355 (8)	0.0025 (6)	0.0065 (6)	0.0062 (6)
O3	0.0586 (9)	0.0640 (10)	0.0373 (8)	−0.0117 (7)	0.0125 (7)	−0.0011 (7)
O4	0.0548 (9)	0.0617 (9)	0.0338 (8)	−0.0022 (7)	0.0066 (6)	0.0060 (6)
O5	0.0446 (8)	0.0472 (8)	0.0383 (8)	−0.0030 (6)	0.0057 (6)	−0.0039 (6)
C1	0.0453 (11)	0.0522 (12)	0.0367 (11)	0.0023 (9)	0.0119 (9)	0.0025 (8)
C2	0.0436 (11)	0.0538 (12)	0.0310 (10)	−0.0062 (9)	0.0050 (8)	0.0003 (8)
C3	0.0411 (10)	0.0519 (11)	0.0311 (9)	0.0003 (9)	0.0048 (8)	0.0023 (8)
C4	0.0392 (10)	0.0520 (11)	0.0321 (10)	−0.0041 (8)	0.0028 (8)	0.0017 (8)
C5	0.0534 (13)	0.0552 (13)	0.0380 (11)	0.0041 (10)	0.0035 (10)	−0.0019 (9)
C6	0.0521 (12)	0.0463 (11)	0.0390 (11)	0.0015 (9)	0.0128 (9)	−0.0016 (8)
C7	0.0511 (12)	0.0557 (12)	0.0344 (10)	−0.0038 (10)	0.0026 (9)	−0.0012 (9)
C8	0.0445 (11)	0.0535 (12)	0.0327 (10)	−0.0027 (9)	0.0019 (8)	−0.0055 (8)
C9	0.0503 (12)	0.0507 (12)	0.0419 (11)	−0.0062 (10)	0.0049 (9)	−0.0031 (9)

### Geometric parameters (Å, º)

**Table:**

O1—C2	1.335 (3)	C3—C4	1.522 (3)
O1—C3	1.451 (2)	C3—C5	1.505 (3)
O2—C1	1.449 (3)	C5—H5A	0.91 (3)
O2—C4	1.344 (3)	C5—H5B	0.98 (3)
O3—C2	1.203 (3)	C5—H5C	0.96 (3)
O4—C4	1.203 (3)	C6—H6A	0.97 (2)
O5—C6	1.421 (3)	C6—H6B	0.99 (3)
O5—C7	1.432 (3)	C7—H7A	0.98 (2)
C1—H1	1.04 (2)	C7—H7B	1.00 (3)
C1—C2	1.515 (3)	C7—C8	1.470 (3)
C1—C6	1.515 (3)	C8—C9	1.184 (3)
C3—H3	0.92 (2)	C9—H9	0.88 (3)
			
C2—O1—C3	119.98 (16)	C3—C5—H5A	110.8 (16)
C4—O2—C1	121.22 (16)	C3—C5—H5B	107.6 (15)
C6—O5—C7	112.74 (16)	C3—C5—H5C	107.4 (16)
O2—C1—H1	104.3 (14)	H5A—C5—H5B	106 (2)
O2—C1—C2	113.46 (17)	H5A—C5—H5C	110 (2)
O2—C1—C6	111.23 (18)	H5B—C5—H5C	115 (2)
C2—C1—H1	106.6 (13)	O5—C6—C1	107.90 (17)
C6—C1—H1	110.0 (13)	O5—C6—H6A	110.3 (13)
C6—C1—C2	110.91 (17)	O5—C6—H6B	111.0 (14)
O1—C2—C1	118.72 (18)	C1—C6—H6A	109.2 (13)
O3—C2—O1	120.44 (19)	C1—C6—H6B	109.7 (14)
O3—C2—C1	120.83 (19)	H6A—C6—H6B	108.8 (19)
O1—C3—H3	109.1 (13)	O5—C7—H7A	108.0 (13)
O1—C3—C4	112.30 (16)	O5—C7—H7B	109.8 (14)
O1—C3—C5	106.96 (18)	O5—C7—C8	112.01 (17)
C4—C3—H3	105.6 (13)	H7A—C7—H7B	109.8 (18)
C5—C3—H3	111.0 (13)	C8—C7—H7A	106.4 (13)
C5—C3—C4	111.88 (17)	C8—C7—H7B	110.8 (13)
O2—C4—C3	117.51 (17)	C9—C8—C7	179.1 (2)
O4—C4—O2	119.26 (18)	C8—C9—H9	177.3 (19)
O4—C4—C3	123.22 (19)		
			
O1—C3—C4—O2	−31.7 (2)	C3—O1—C2—O3	168.81 (18)
O1—C3—C4—O4	149.05 (19)	C3—O1—C2—C1	−11.5 (3)
O2—C1—C2—O1	−23.6 (3)	C4—O2—C1—C2	30.6 (3)
O2—C1—C2—O3	156.06 (19)	C4—O2—C1—C6	−95.2 (2)
O2—C1—C6—O5	70.2 (2)	C5—C3—C4—O2	−152.00 (19)
C1—O2—C4—O4	176.43 (18)	C5—C3—C4—O4	28.8 (3)
C1—O2—C4—C3	−2.8 (3)	C6—O5—C7—C8	71.3 (2)
C2—O1—C3—C4	39.0 (2)	C6—C1—C2—O1	102.4 (2)
C2—O1—C3—C5	162.14 (17)	C6—C1—C2—O3	−77.9 (2)
C2—C1—C6—O5	−57.0 (2)	C7—O5—C6—C1	−174.10 (17)

### Hydrogen-bond geometry (Å, º)

**Table:**

*D*—H···*A*	*D*—H	H···*A*	*D*···*A*	*D*—H···*A*
C3—H3···O4^i^	0.92 (2)	2.60 (2)	3.247 (3)	127.8 (16)
C3—H3···O5	0.92 (2)	2.49 (2)	3.033 (2)	118.1 (16)
C6—H6*B*···O3^ii^	0.99 (3)	2.47 (2)	3.369 (3)	151.0 (19)
C7—H7*A*···O3^iii^	0.98 (2)	2.66 (2)	3.627 (3)	169.1 (18)
C9—H9···O4^iv^	0.88 (3)	2.58 (3)	3.412 (3)	156 (2)

Symmetry codes: (i) −*x*+1, −*y*+1, −*z*+1; (ii) −*x*+2, *y*−1/2, −*z*+1/2; (iii) *x*−1, *y*, *z*; (iv) −*x*+1, −*y*, −*z*+1.
