# Supplementary material for: Synthesis and characterization of 3-methyl-6-[(propyn­yloxy)meth­yl]-1,4-dioxane-2,5-dione
Source: Acta Crystallogr E Crystallogr Commun. 2017 Jun 16;73(Pt 7):1044–7. doi: 10.1107/S2056989017008581 (PMC5499287; doi:10.1107/S2056989017008581)
Supplement: Supplementary file 3 [file e-73-01044-sup3.pdf]

# Supporting Information

## Synthesis and characterization of 3-methyl-6-propynyloxymethyl-1,4-dioxane-2,5-dione

Igor Elkin,[a] Thierry Maris[b] and Patrice Hildgen[a]*

[a]Faculty of Pharmacy, Université de Montréal, 2900 Edouard-Montpetit Blvd, Montreal, Quebec, H3T1J4, Canada, and [b]Department of Chemistry, Université de Montréal, 2900 Edouard-Montpetit Blvd, Montreal, Quebec, H3T1J4, Canada

Correspondence e-mail: patrice.hildgen@umontreal.ca

**Details related to the synthesis, purification and characterization techniques used in the study**

## *1. Chemicals*

Nitric acid (68-70w/w% A200-212, certified ACS plus), magnesium sulfate (anhydrous certified powder), diethyl ether (certified ACS), hexanes (various methylpentanes, certified ACS, 99.9%) were purchased from Fisher Chemical (Ottawa, ON, Canada). (±)3-Chloro-1,2-propanediol (98%), sodium bicarbonate (cell culture tested), sodium hydride (60% dispersion in mineral oil), tetrahydrofuran (anhydrous, ≥99.9%, inhibitor free), propargyl alcohol (99%), 2-bromopropyonyl bromide (97%), methylene chloride (≥99.5%) were provided by Sigma-Aldrich (Oakville, ON, Canada). Triethylamine (99.7%) and acetonitrile (≥99.9%, extra dry, over molecular sieve, Acros Sieve) were purchased from Acros Organic (Fair Lawn, NJ, US).

## *2. Characterization techniques (other than X-Ray)*

*Fourier Transform Infrared Spectroscopy (FTIR).* All FTIR measurements were performed on a Nicolet iS10 purchased from Thermoscientific Co. (Madison, WI, US) with attenuated total reflectance (ATR) sampling technique (optical material: ZnSe, number of scans: 64, acquisition interval: 0.482 cm-1) with a spectral interval of 4000 to 500 cm-1, using OMNIC 8.1.10 (Thermoscientific Co.) software to analyse obtained spectra.

*Nuclear Magnetic Resonance (NMR).* Unidimensional 1H and 13C NMR spectra were recorded on a Bruker AMX-300 (Karlsruhe, Germany) with a 5-mm dual proton/carbon probe at a frequency of 300.03 MHz for 1H, and 75 MHz for 13C respectively, at a maximal magnetic induction of 7.01 T, using CDCl3 as solvent in all experiments. Bidimensional 1H/1H NOESY spectra were measured with Bruker Avance 300 (Karlsruhe, Germany) equipped with 5 mm INVERSE probe at 300.03 MHz. All spectral analyses were done with software MestRec4.9.9.6 from Mestrelab Research (Escondido, CA, US).

*Elemental Analysis of N, C, H, and S* (EA) was carried out on a Fisons EA 1108 from Fisons Instruments (Rodano, Italia) and a Costech 4010 from Isomass Co. (Valencia, CA, US) analyzers by flash dynamic combustion technique. The analysis of the chromatograms obtained for combustion products of analyzed organic samples (N2, CO2, H2O, and SO2) was performed using Eager 300 software from Thermo Electron Co. (Madison, WI, US).

*Liquid Chromatography - Mass Spectrometry (LC-MS).* The chromatograms and mass-spectra were obtained on a LC-MS spectrometer from Agilent Technologies 1260 Infinity (Santa Clara, CA, US) in ESI positive and negative modes. The separation was performed on a Agilent Poroshell 120 EC-C18 2.7 μm, using acetonitrile-water-acetic-acid mixtures (Solvent A: water/acetonitrile (95%/5%) + acetic acid (0.1%); Solvent B: acetonitrile (100%) + acetic acid (0.1%)) in gradient elution mode at a flow rate of 1.5 mL/min. The detection was carried out with both, Agilent 1260 Infinity Multiple Wavelength Detector G1356C and Single Quadrupole

LC/MS 6120 detector, using ESI source operated in positive or negative ion modes, at a capillary voltage of 1000 V, a vaporizer temperature of 150°C, a nebulization pressure of 60 psig, a dry gas temperature of 300°C, and at a gas flow of 5 L/min. The scanning was done from 100 to 700 Da/z. The spectral analysis was performed with Agilent OpenLAB CDS Chemstation Edition for LC and LC/MS systems REV: C.01.06-75 (Agilent Technologies).

*Flash-chromatography purification technique.* Purification of crude products using the flash-d chromatography purification technique, was performed on Combi Flasf Rf 150 (Teledine ISCO, Lincoln, Nebraska, USA) with SiliaSep (40g, FLH-R10030B-ISO40) and SiliaSep C18 (40g, FLH-R33230B-ISO40) flash-cartridges provided by SiliCycle Inc. (Quebec, QC, Canada).

### *3. Chemical synthesis, purification and structural characterization of products*

*(±)3-Chloro-2-hydroxypropanoic acid (**3**).*

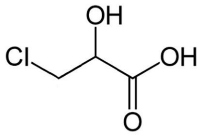


100 g (0.905 mol) of (±)3-chloro-1,2-propanediol and 300 mL of 70%-aqueous nitric acid were mixed at r.t. After reaching gradually 105°C by an external heating (CAUTION! At about 60°C a vigorous reaction starts and the evolution of a brown gas with an increase in t°C should be constantly monitored. In our case, the gas was neutralized chemically by bubbling into 3L of 3%-7% aqueous NaOH under a fume hood), the mixture was stirred for 5 h. After completion, the residual nitric acid was gradually neutralized with 117 g (1.405 mol) $NaHCO_3$ (CAUTION! An intensive $CO_2$-formation!) followed by evaporating water under reduced pressure and finally, by extracting **3** with diethyl ether in the presence of $MgSO_4$ and drying under vacuum, to give a white solid. Yield: 57.5 g (51.07 %). $^1$H NMR (δ, ppm; 300 MHz; DMSO-*d*6): 6.00-5.40 (m, 1H, -C(O)O*H*), 4.4-4.2 (m, 1H, -C(OH)*H*-), 3.8-3.6 (m, 2H, -C*H*2-Cl), 3.6-3.0 (m, 1H, -C(O*H*)H-). $^{13}$C NMR (δ, ppm; 100 MHz; DMSO-*d*6): 172.9 (-*C*(O)OH), 70.5 (-*C*$H_2$-), 47.6 (Cl-*C*-). FTIR ($\nu_{min}$, cm$^{-1}$): 3320 (C(O)O-H, O-H), 2982, 2938, 2916 (C-H), 1766, 1740, 1708 (-O-C(O)-), 1464, 1436, 1411, 1375 (>$CH_2$), 1299, 1209, 1178, 1152, 1090 (C-O), 1002, 917, 869, 804, 773, 758 (C-Cl), 674. MS (m/z): EM calculated for $C_3H_5O_3Cl$: 124.0 $[M]^0$; found by ESI (-): 123.1 $[M-H]^-$, 247.0 $[2M-H]^-$. See also Figures SI-1-3.

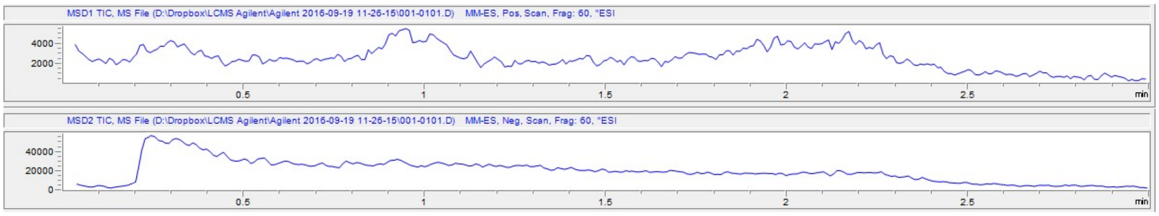


**a.**

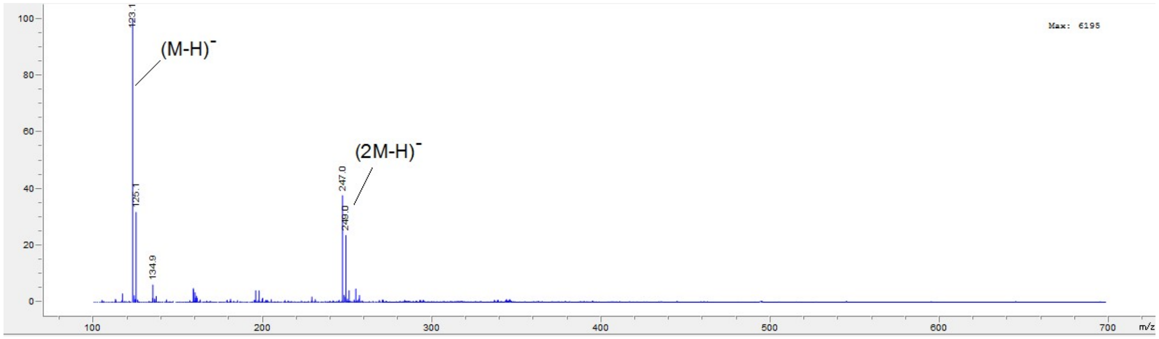


**b.**

**Figure SI-1. LC-MS analysis of 3-Cl-2-hydroxypropanoic acid (3): a. chromatograms of the product obtained in positive (top) and negative mode (bottom); mass spectrum by ESI in negative mode.**

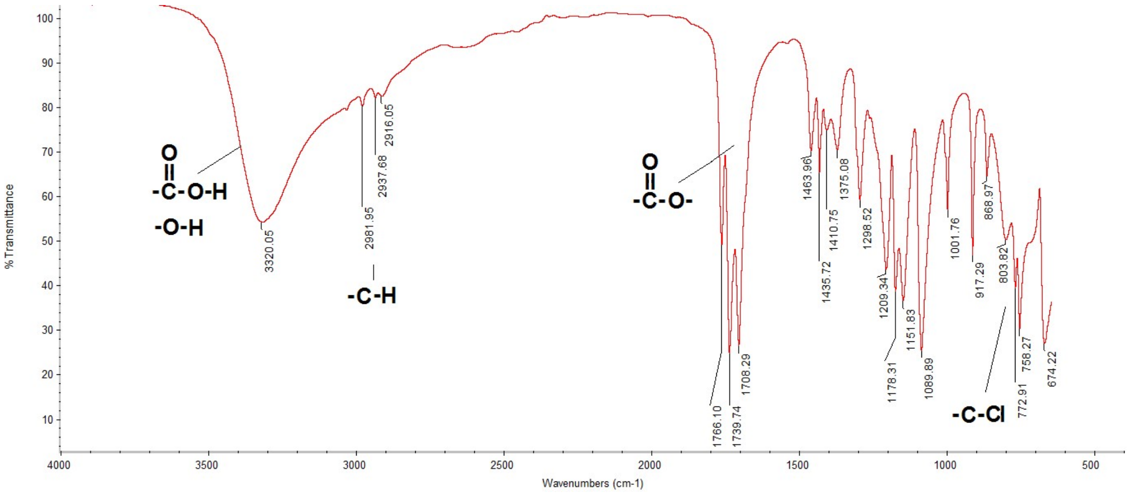


**Figure SI-2. FTIR spectrum of 3-Cl-2-hydroxypropanoic acid (3).**

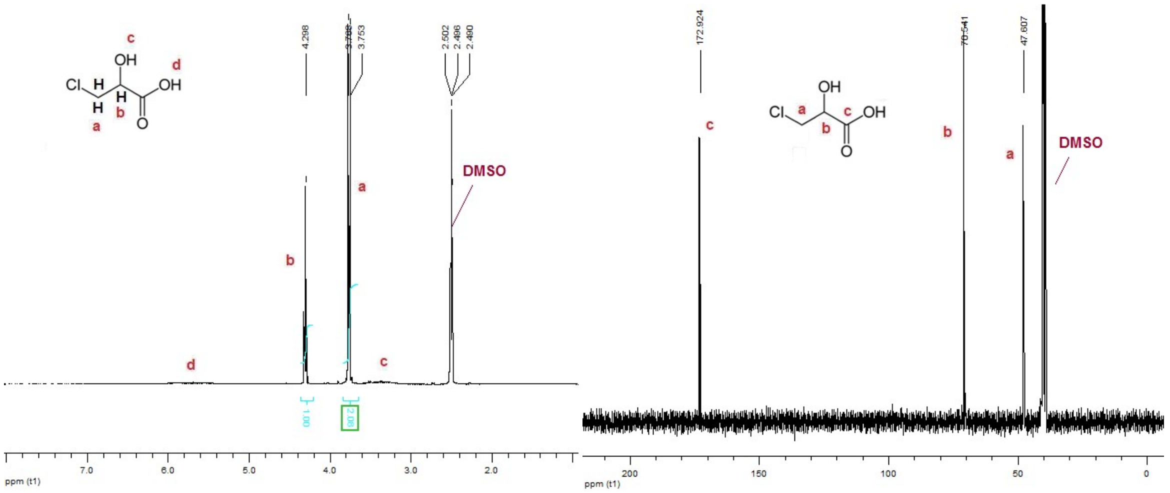


**a.** **b.**

**Figure SI-3.** 1**H (a) and 13C (b) NMR spectra (DMSO-d6) of 3-Cl-2-hydroxypropanoic acid (3).**

*1-Propynyloxymethyl-2-hydroxypropanoic acid (**4**).*

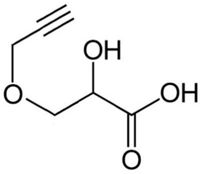


55 g (0.44 mol) of **3** were solubilised in 400 mL of dry THF and after cooling to 0ºC, 18 g of 60% NaH (0.45 mol) in mineral oil were gradually added, and the resulting mixture was stirred for 1h under argon atmosphere. In parallel, 45 g (0.8031 mol) of propargyl alcohol were reacted with 18 g of 60% NaH (0.45 mol) in mineral oil at 0ºC over 1h under argon atmosphere, using 400mL of dry THF as a medium. Both suspensions were mixed at r.t., and the resulting mixture was heated at 105 ºC at reflux for approximately 24h (a supplementary manual shaking of the reaction flask, i.e., once per hour, was necessary for an effective homogenizing). After completion (monitored by MS-ESI, till the absence of signals of the initial **3**), the solvent was removed under reduced pressure and the residue was solubilised in 500 mL of water. Non-polar side ingredients were extracted with diethyl ether and the residual dark aqueous phase was acidified to pH 1.1 with HClaq. The obtained mixture was treated with active carbon at 95 ºC for 1.5 h and then filtered to give a clear yellowish solution which was evaporated under vacuum. The dry residue was extracted first with 50 mL of $CH_2Cl_2$ to remove impurities and then with 500 mL of diethyl ether in the presence of $MgSO_4$ to extract and dry the crude **4**. After filtration and evaporating solvent, the pure product was obtained by recrystallization from the mixture $CH_2Cl_2$-hexane (4:1). Yield: 32.2 g (50.8% 100% is 63.417g). $^1H$ NMR (δ, ppm; 300 MHz;

DMSO-*d6*): 6.00-5.60 (m, 1H, -C(O)O*H*), 4.5-4.0 (m, 1H of -C(OH)*H*- and 2H of –C$H_2$-C≡), 3.8-3.5 (m, 2H, CH(OH)-C$H_2$-O-), 3.5-3.0 (m, 1H of ≡C-*H* and 1H of -C(O*H*)H-). $^{13}$C NMR (δ, ppm; 100 MHz; DMSO-*d6*): 174.0 (-*C*(O)OH), 80.6 (-*C*≡CH), 77.7 (-C≡*C*H), 71.8 (-*C*(OH)H-), 70.2 (-C(OH)H-*C*$H_2$-), 58.3 (-O-*C*$H_2$-C≡). FTIR ($\nu_{min}$, cm$^{-1}$): 3293 (C(O)O-H, O-H), 3258 (≡C-H), 2958, 2942, 2915 (C-H), 2643, 2118 (-C≡C-), 1718 (-O-C(O)-), 1459, 1439, 1422, 1382 (>$CH_2$), 1330, 1279, 1240, 1132, 1078, 1035 (C-O), 1022, 927, 878, 824, 779, 717, 681. MS (m/z): EM calculated for $C_6H_8O_4$: 144.1 $[M]^0$; found by ESI-QP (-): 143.1 $[M-H]^-$, 287.0 $[2M-H]^-$. See also Figures SI-4-6.

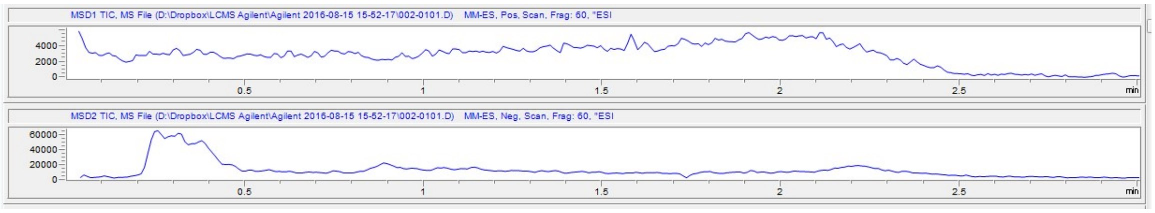

**a.**

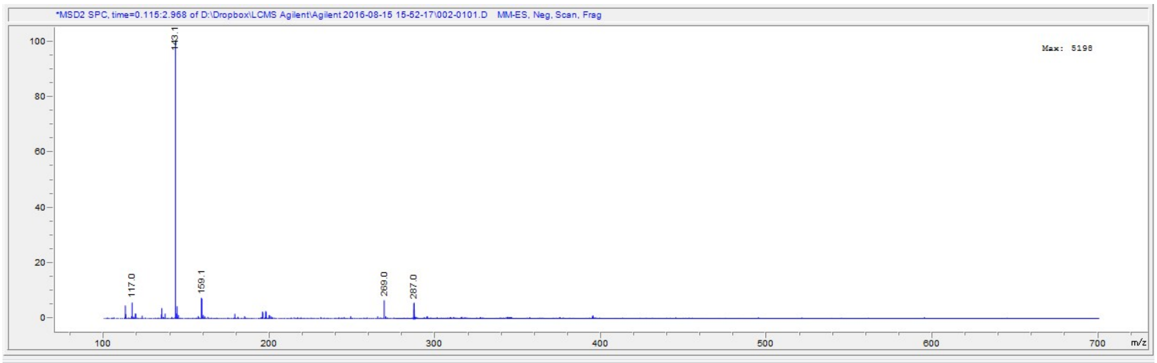


**b.**

**Figure SI-4. LC-MS analysis of 1-propynyloxymethyl-2-hydroxypropanoic acid (4) represented by colorless crystals obtained after recrystallization: LC-MS chromatogram (a); MS (ESI, negative) spectrum (b).**

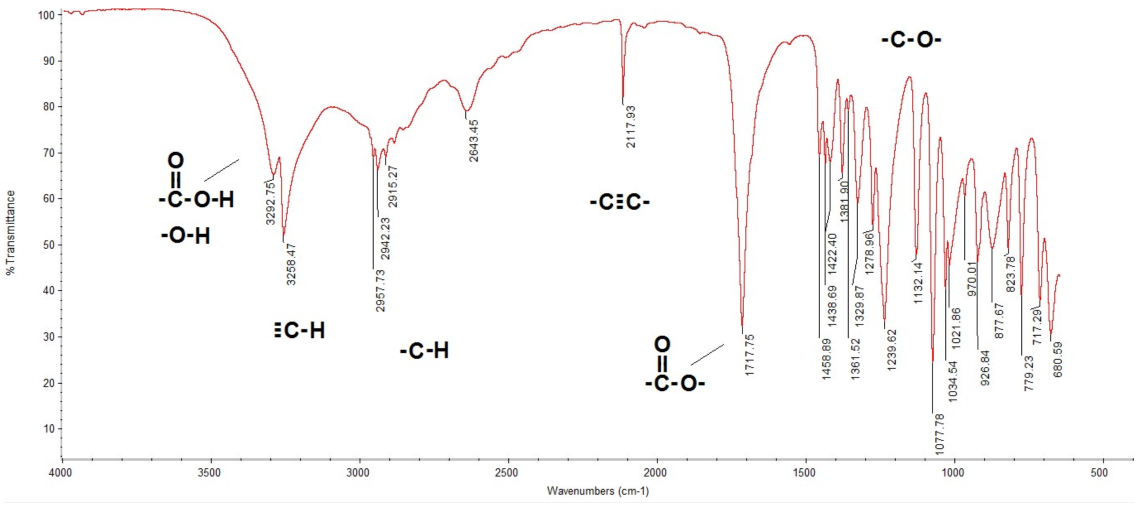


Figure SI-5. FTIR spectrum of 1-propynyloxymethyl-2-hydroxypropanoic acid (4) purified by recrystallization technique.

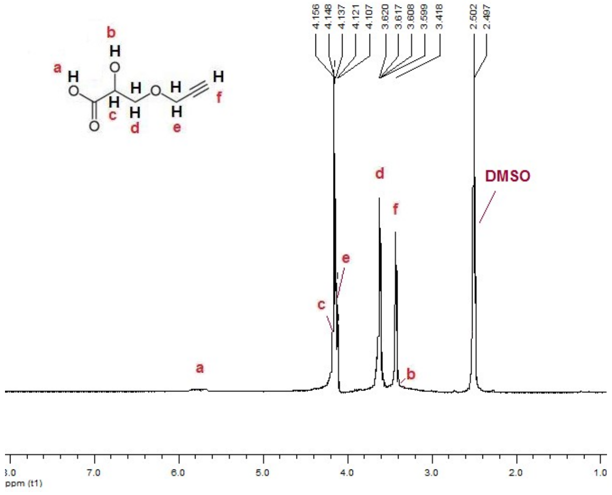


a.

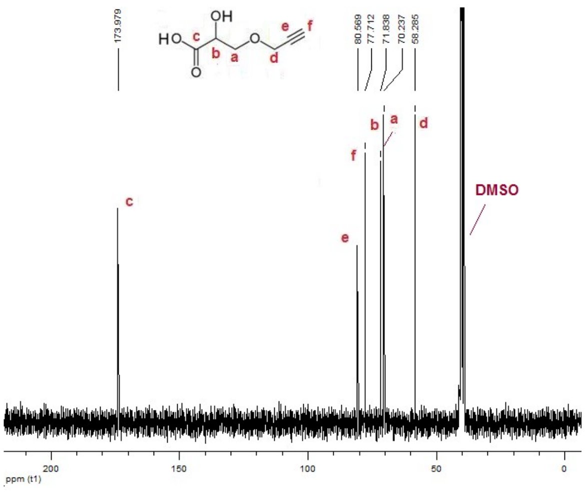


b.

Figure SI-6. 1H (a) and 13C (b) NMR spectra (DMSO-d6) of 1-propynyloxymethyl-2-hydroxypropanoic acid (4) purified by recrystallization technique.

*3-Methyl-6-propynyloxymethyl-1,4-dioxane-2,5-dione (**1**).*

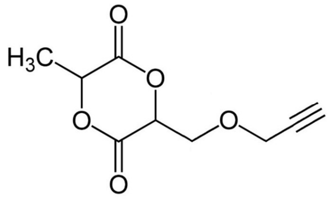

0.25 g (0.0017 mol) of freshly prepared **4** were solubilized in 30 mL of dry MeCN and after cooling to 0°C, first, 0.5 mL g (0.0034 mol) of triethylamine and then 0.18 mL (0.0017 mol) of bromopropyonyl bromide were added. the resulting mixture was stirred under argon atmosphere first at r.t. for 3 h, then, at 50°C for 2h and finally, at 70°C for 2h. After completion, the reaction mixture was evaporated under reduced pressure and the crude product was extracted with $CHCl_3$. The solution was washed first with 1M HCl (2x50 mL), then with 10%-aqueous NaCl (1x50mL), and dried with $MgSO_4$. The final purification was performed by flesh-chromatography on silica, using chloroform as eluent to give a white solid. Yield: 0.07 g (20%). $^1$H NMR (δ, ppm; 300 MHz; $CDCl_3$): 5.4-5.2 (m, 1H, >C*H*-$CH_2$-O-), 5.2-5.0 (m, 2H, >C*H*-$CH_3$), 4.26-4.18 (m, 2H, -O-C*H*2-C≡), 4.18-3.9 (m, 2H, >CH-C*H*2-O-), 2.6-2.4 (m, 1H, -C≡C-*H*), 1.7-1.5 (m, 3H, -C*H*3). $^{13}$C NMR (δ, ppm; 100 MHz; $CDCl_3$): 166.5, 165.4 (-O-C($CH_3$)-*C*(O)O-), 77.5, 77.2 (-C≡*C*H and -*C*≡CH), 76.3 (>*C*H-$CH_2$-O-), 72.6 (>*C*H-$CH_3$), 70.5 (>CH-*C*(O)O-), 58.9 (>CH-*C*$H_2$-O- and -O-*C*$H_2$-C≡), 17.4 (-*C*$H_3$). FTIR ($\nu_{min}$, cm$^{-1}$): 3271 (≡C-H), 3012, 2976, 2956, 2940, 2915, 2885, 2867 (C-H), 2118 (-C≡C-), 1748 (-O-C(O)-), 1465, 1444, 1380 (>CH-), 1344, 1322, 1305, 1274, 1256, 1232, 1203, 1126, 1094, 1055, 1035 (C-O), 986, 960, 921, 831, 801, 770, 730, 701, 683. MS (m/z): EM calculated for $C_9H_{10}O_5$: 198.1 $[M]^0$; found by ESI-QP (-): 195.7 $[M-2H]^-$, 197.7 $[M]^-$, 201.8 $[M+3H]^-$, 215.0 and 215.9 $[M+H_2O-H]^-$. EA (%): calculated, %: C 54.49, H 5.45; found, %: C 54.14, H 5.08. See also Figures SI-7-9, as well as the main text related to the X-Ray diffraction analysis of the product.

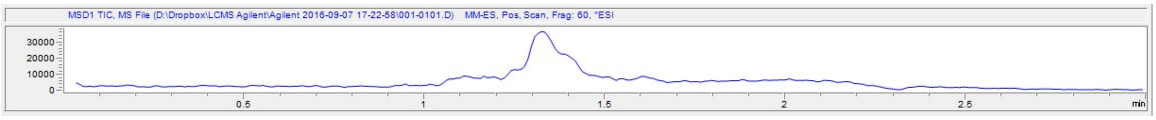


**a.**

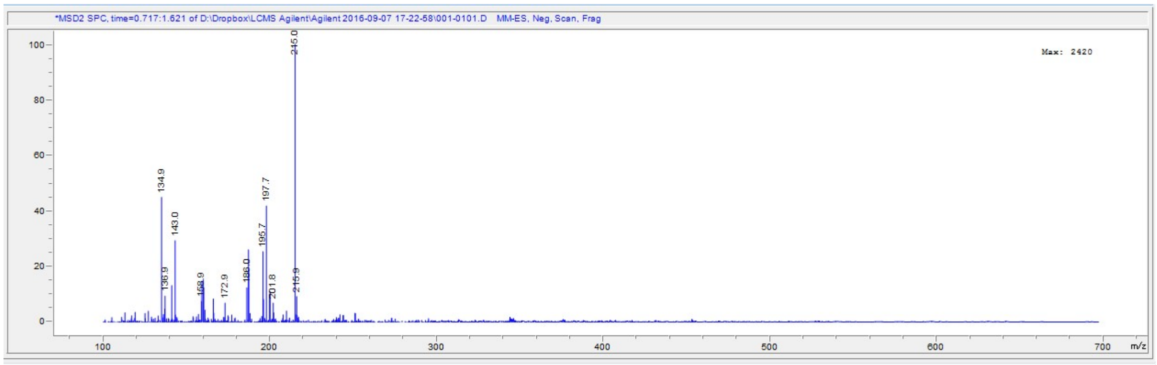


**b.**

**Figure SI-7. LC-MS analysis of 3-methyl-6-propynyloxymethyl-1,4-dioxane-2,5-dione (1) represented by colorless crystals obtained after purification by chromatography (silica, CHCl3): LC-MS chromatogram (a); MS (ESI, negative) spectrum (b).**

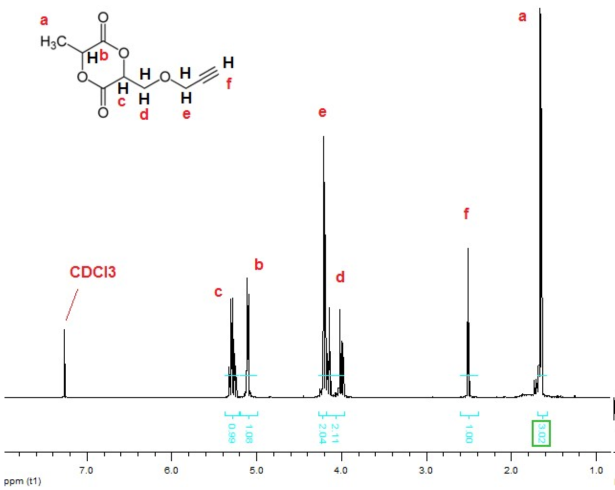


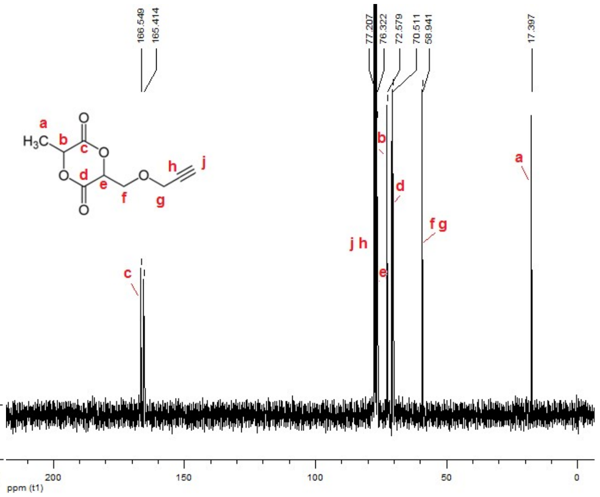


**a.** **b.**

**Figure SI-8. 1H (a) and 13C (b) NMR spectra (CDCl3) of 3-methyl-6-propynyloxymethyl-1,4-dioxane-2,5-dione (1).**

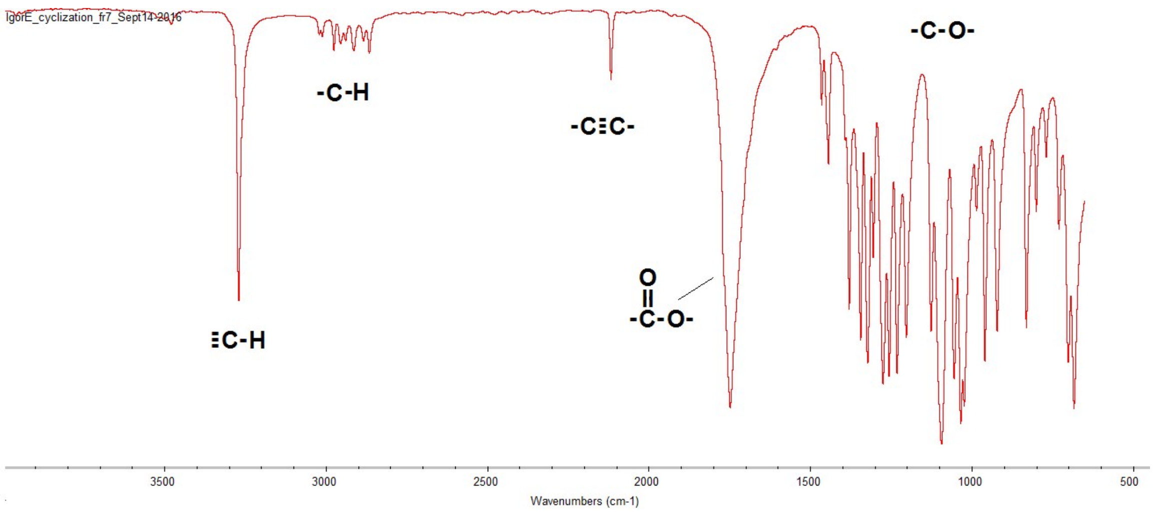


**Figure SI-9. FTIR spectrum of 3-methyl-6-propynyloxymethyl-1,4-dioxane-2,5-dione (1).**
